# Permeability selection of biologically relevant membranes matches the stereochemistry of life on Earth

**DOI:** 10.1371/journal.pbio.3003155

**Published:** 2025-05-20

**Authors:** Olivia Goode, Urszula Łapińska, Juliano Morimoto, Georgina Glover, David S. Milner, Alyson E. Santoro, Stefano Pagliara, Thomas A. Richards

**Affiliations:** 1 Living Systems Institute and Biosciences, University of Exeter, Exeter, United Kingdom; 2 Department of Biology, University of Oxford, Oxford, United Kingdom; 3 Institute of Mathematics, University of Aberdeen, King’s College, Aberdeen, United Kingdom; 4 Programa de Pós-graduação em Ecologia e Conservação, Universidade Federal do Paraná, Curitiba, Brazil; 5 Department of Ecology, Evolution and Marine Biology, University of California, Santa Barbara, California, United States of America; University College London, UNITED KINGDOM OF GREAT BRITAIN AND NORTHERN IRELAND

## Abstract

Early in the evolution of life, a proto-metabolic network was encapsulated within a membrane compartment. The permeability characteristics of the membrane determined several key functions of this network by determining which compounds could enter the compartment and which compounds could not. One key feature of known life is the utilization of right-handed d-ribose and d-deoxyribose sugars and left-handed l-amino acid stereochemical isomers (enantiomers); however, it is not clear why life adopted this specific chirality. Generally, archaea have l-phospholipid membrane chemistries and bacteria and eukaryotes have d-phospholipid membrane chemistries. We previously demonstrated that an l-archaeal and a d-intermediate membrane mimic, bearing a mixture of bacterial and archaeal lipid characteristics (a ‘hybrid’ membrane), displayed increased permeability for several key compounds compared to bacterial-like membranes. Here, we investigate if these membranes can drive stereochemical selection on pentose sugars, hexose sugars, and amino acids. Using permeability assays of homogenous unilamellar vesicles, we demonstrate that both membranes select for d-ribose and d-deoxyribose sugars while the hybrid membrane uniquely selects for a reduced alphabet of l-amino acids. This repertoire includes alanine, the plausible first l-amino acid utilized. We conclude such compartments could provide stereochemical compound selection matching those used by the core metabolism of life.

## Abstract 2

Early in the evolution of life a biochemical network was encapsulated within a membrane compartment. This study shows that specific membrane forms can select for stereochemical compounds which match the pattern of stereochemistry used by all known living things.

## Introduction

Life on Earth is defined by a curious and universal stereochemical asymmetry [1,2]. Specifically, the pentose sugars utilized for DNA and RNA (deoxyribose and ribose) possess three stereocenters imposing a d- (right-handed) stereochemistry. In contrast, 19 of the 20 universal proteinogenic amino acids possess one or two stereocenters imposing l- (left-handed) stereochemistry on polypeptides. A number of abiotic processes have been proposed for generation of homochiral states (e.g., [1–6]), while other works suggest stereoselectivity for the transfer of l-configured aminoacyl residues in tRNA [7–10]. Importantly, autocatalytic polymerization of mononucleotides proceeds with efficacy in mixtures with high relative ratios of one enantiomer [11]. Similar catalytic processes have been demonstrated for amino acids, which can polymerize stereo-selectively in abiotic conditions [3,12]. These data demonstrate Wald’s conjecture [1], which set out how the polymerization characteristics of ribonucleotides and amino acids would lead life to preferentially adopt a single stereochemical form. While these data explain why life might use homochiral states, they do not explain the conditions under which specific homochiral mixes arose in a biologically proximate system.

Numerous ideas about the origin of life have been influenced by the demonstration that abiotic chemical conditions can generate many compounds utilized by living systems (see [13]). Experimentally simulated prebiotic conditions have been shown to form ribose through the formose reaction [14], while a single abiotic phosphorylation mechanism can account for the phosphorylation reactions found across core metabolism [15], including the production of ribose-5-phosphate. The distribution of variant glycolysis pathways across the tree of life [16,17] also suggests that glycolysis was an ancestral feature of the last universal common ancestor (LUCA) [18,19]. Consistent with the phylogenetic age of these pathways, experimental chemical simulations of the early Earth ocean led to the formation of glycolysis intermediates [20], suggesting glycolytic pathway compounds were available for catabolism and as precursors of both ribonucleotides and phospholipids. Additional work has suggested that gluconeogenesis preceded glycolysis [21] and, therefore, glucose, and possibly other hexose sugars, were available for early life forms. Similarly, the Miller experiments [22,23] demonstrate that a subset of amino acids can form in simulated early Earth conditions. These experiments demonstrate glycine and alanine are generated at much higher concentrations than any other proteinogenic amino acid. Furthermore, alanine has been shown to form abiotically via transamination of oxaloacetate when exposed to a diverse range of cation catalysts [24]. A similar amino acid composition to those recovered in the Miller experiments has been detected in meteorite-derived materials [25]. These meteorite data provide important ‘outside-the-lab’ demonstrations that chemical compounds relevant for proto-metabolic function can form in abiotic conditions.

A systematic review of 40 different criteria has been used to reconstruct the relative temporal order of amino acid utilization as life evolved, providing a consensus that glycine and alanine were the first amino acids utilized [26,27]. Glycine does not possess any stereocenters, while alanine has one. As such, the use of l-alanine over d-alanine putatively imposed l-stereochemistry on all future biogenic proteins. We note that if life started with glycine and alanine, as implicated by the Miller experiments [22,23] and many reconstructions of the evolution of the genetic code [26–28], propagation of chiral selection would be at the level of the polymerization of the amino acid peptide as a wider diversity of amino acids were incorporated. This is because amino acid chirality is not propagated through biosynthetic networks of known amino acid precursors (e.g., the reverse Krebs cycle). Yet, the amino acids generated from the Miller experiments and detected in meteorite samples are racemic, possessing equal shares of both enantiomers [22,25], a mixture of compounds that would curb amino acid polymerization and the evolution of early metabolic functions [1,3,12] without enantiomer selection.

J. B. S. Haldane discussed how life could have emerged ‘enclosed in an oily film’ [29], a scenario which would have separated early biological systems from abiotic mixtures present in the surrounding environment. Independent of whether life started as a heterotrophic form [30,31] (i.e., the ‘Oparin–Haldane’ theory [32]) or an autotrophic form [20,28,33–35], a critical step in the history of life involved the encapsulation of metabolic functions within a membrane [36,37]. Acquisition of a membrane bestowed a proto-cellular form with the capacity for metabolite accumulation [38], generation of chemical gradients [39], and co-association of interacting systems necessary for co-function [37]. Compartmentalization also generated divisible units, allowing variation in biochemical functions including different reaction kinetics, upon which Darwinian selection could act [40,41]. Cell membranes have been known to be semi-permeable since the late 18th Century [42,43], forming a barrier that can selectively allow some compounds to pass through while excluding others (e.g., [44,45]). The permeability of proto-cellular membranes must therefore have determined the degree to which internal membrane space is a chemically distinct environment [38,46,47].

Fatty acid membrane compartments of various compounds are useful model systems [48] for understanding protocell evolution. For example, they have been shown to assemble from meteorite extracts [49] and can be generated from clays in a process which can encapsulate RNA templates [50]. Such membranes are also permeable to chemical precursors necessary for core cellular functions [30,38,47,51] and can ‘grow’ in a process driven by vesicle content [40,52] including internalized RNA replication [30]. Nonetheless, there are chemical limitations to fatty acid membranes as proto-cellular systems; chiefly, such vesicles cannot maintain integrity in environments with high concentrations of divalent cations (e.g., seawater) [41]. Similarly, they cannot host the high concentrations of Mg^2+^ thought necessary for ribozyme-catalyzed polymerase function, which has been suggested to act as an early replicator in the RNA world hypothesis [30,53–57]. These observations suggest that a phase of proto-cellular evolution using an alternative membrane chemistry was necessary.

The membrane of all extant cellular life is built from variant phospholipids [58]. Experiments have demonstrated that vesicles of eukaryotic membrane analogs (POPC and DPPC) are permeable to a range of pentose sugars including ribose [47], while vesicles of both bacterial and eukaryotic membrane models (EPC, DMPC, DOPC/POPC, and DPPC) are permeable to amino acids [44–46]. Phospholipid vesicles can encapsulate macromolecules upon dehydration–hydration cycles [59], making them important models for understanding how proto-cellular compartments could internalize metabolic compounds, templates or catalysts. Furthermore, phospholipids can form in abiotic conditions [60], can be used to encapsulate RNA polymerization [61], can perform budding and division in the presence of ad hoc precursors [62], and can withstand ion concentration gradients [63].

A range of phospholipid forms are used by microbes, often in heterogenous and varying mixes, sometimes with natural cells and synthetic manipulated systems possessing membranes combining different stereochemical forms [64–66]. Despite this diversity, there are two fundamental core membrane phospholipid chemistries present in cellular life [58]: bacteria and eukaryotes possess membranes predominantly composed of fatty acid chains bonded to a glycerol-3-phosphate (G3P) backbone via ester bonds; a phospholipid with a right-handed stereochemistry. In contrast, many archaeal membranes are predominantly constructed from diether lipids with isoprenoid chains containing methyl branches bonded to a glycerol-1-phosphate (G1P) backbone via ether bonds; a phospholipid with a left-handed stereochemistry [58,63,67]. This pattern demonstrates that deep within the tree of life two different lipid forms were adopted on distinct branches [58] (this is known as the lipid-divide). It is not clear if this transition was from: (i) one form to the other, (ii) an intermediate form (for example, a phospholipid with a combination of bacterial and archaeal characteristic, i.e., a ‘hybrid’ membrane), (iii) the fatty acid membranes similar to the types discussed above, (iv) an ancestral cell possessing a mixed heterochiral membrane (e.g., [64–66]), or (v) an intermediate life form that possessed no membrane; with the two membrane types adopted at later stages, independently [34,68,69]. There is currently limited consensus on the placement of LUCA that would enable us to polarize the transition in membrane chemistry. Some analysis suggests that the root of the tree of life may reside between the Bacteria and the Archaea [70–75], while alternative analyses suggest that the root lies within the Bacteria [76–78]. The placement of the root determines the minimum number and nature of transitions in membrane chemistry during the evolution of the prokaryotic domains. Neither rooting scenario excludes the presence of hybrid or mixed membrane variants deep within either the Bacteria or the Archaea or at their common ancestor. However, we note that if a hybrid membrane was an intermediate form in the evolution of variant membrane compositions, it would be a more parsimonious solution if LUCA were placed between the Bacteria and the Archaea; involving fewer changes in chemical characteristics between the hybrid membrane and the extant membrane phospholipids defined by the lipid divide.

Using unilamellar vesicle microfluidic permeability assays, we have previously shown that archaea-like G1PC diether isoprenoid phospholipid membranes demonstrate higher permeability to core metabolites than bacterial-like G3PE and G3PG diester fatty acid membranes. These core metabolites include: nucleobases (that possess no stereochemistry), ribose, deoxyribose, and six proteinogenic amino acids [79]. These experiments also included comparisons with a plausible intermediate ‘hybrid’ phospholipid membrane consisting of diether lipids with isoprenoid chains containing methyl branches (archaeal traits) bonded to a G3P with right-handed stereochemistry (bacterial traits). Experiments using this hybrid membrane demonstrated increased permeability compared to both bacterial and archaeal membranes for deoxyribose, ribose, and glycine. Alternative hybrid membrane forms with different combinations of chemical characteristics showed reduced permeability in comparison to both the hybrid and archaeal membrane. We therefore suggest that the permeability characteristics of archaeal-like or hybrid membranes might have driven the early evolution of proto-cellular metabolism [79].

Historically, few experiments have compared l- and d-enantiomer permeability through membranes. For example, Nagano in 1902 [80,81] compared the absorption rate of a range of sugars across the intestinal membrane of dogs, an experiment that included comparisons of d-hexose and l-pentose sugars. More recently, Sacerdote and Szostak compared absorption of l- and d-xylose through four homogeneous vesicle types, including three different fatty acids and one phospholipid (POPC, a compound similar to eukaryotic membranes) [47]. This experiment demonstrated no difference in the l- versus d-enantiomer permeability of xylose. Analysis of DOPC/POPC [44] and DPPC [45] membranes using microfluidic droplet interface bilayers showed l-enantiomer selection for a subset of amino acids, with a range of sidechains, over >15 h [44]. A further study showed a weak level of preferential permeation for l-alanine over d-alanine (1.2-fold increase), along with stronger selection for other amino acids, across DOPC/POPC membranes on a much shorter time scale [45]. Here, we expand such comparisons to a range of compounds useful for proto-metabolism and focus on previously ignored archaeal-like and hybrid diether isoprenoid phospholipid membrane mimics. We focus on these two membrane mimics because our previous experiments show they display the highest permeability and because both the fatty acid chain and diester chemical characteristics of eukaryotic and bacterial membranes act to limit permeability [79]. The aim of this work is to test the hypothesis that membrane chemical characteristics impose permeability selection on enantiomers. We compare the enantiomer permeability of four pentose sugars, two hexose sugars, and 12 amino acids, demonstrating strong selection for d-ribose and d-deoxyribose in both the diether isoprenoid membrane types tested but no difference in permeability in the bacterial type diester fatty acid membranes. In contrast, only the hybrid membrane shows permeability selection for multiple l-amino acids, including strong selection for l-alanine, the candidate first chiral amino acid used [26,27]. We, therefore, suggest that early utilization of a ‘hybrid’ membrane chemistry—a possible precursor form to either, or both, bacterial or archaeal membrane chemistry—or an alternative unidentified membrane form with similar permeability characteristics, could have allowed enrichment of specific enantiomers and therefore hardwired life to utilize l-amino acids and d-ribonucleotides.

## Results

### Pentose sugar permeability shows selection for d-ribose and d-deoxyribose

**Fig 1:**
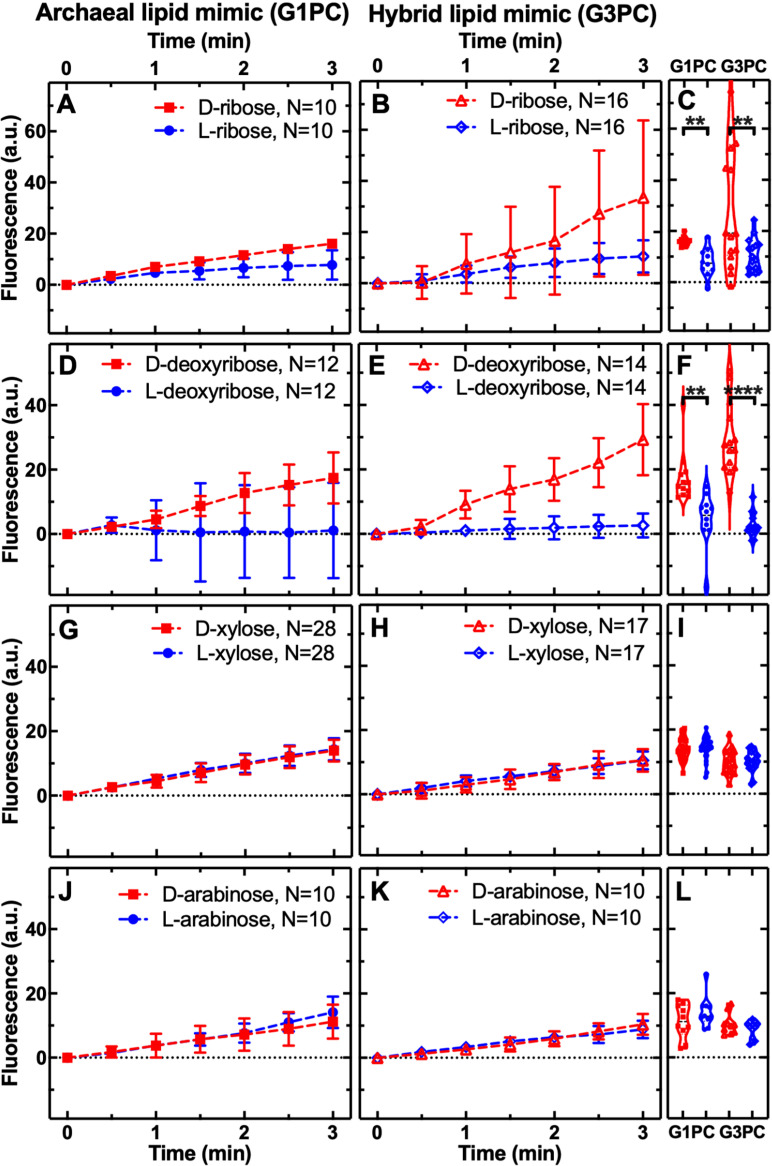
Membrane permeability to pentose sugars shows bias for **d****-ribose and** **d****-deoxyribose.** Temporal dependence of average carboxyfluorescein (CF) fluorescence in the archaea-like G1PC phospholipid membrane (**A, D, G, J**: G1PC, filled symbols) and in the ‘hybrid’ phospholipid membrane (**B, E, H, K**: G3PC, open symbols) during exposure to 1 mM of d- or l-ribose, deoxyribose, xylose or arabinose delivered to the microfluidic coves at a constant rate. Mean (symbols) and standard deviation (error bars) were calculated from at least 10 single-vesicle measurements across three independent experiments. Lines connecting points are provided as a guide. *N* is the number of single vesicles investigated for each substrate exposure and each type of vesicle. *N* varies across different substrate experiments investigated due to technical constraints. However, care has been taken to obtain the same *N* for each substrate experiment across the two different enantiomer treatments in order to ensure reliable statistical comparisons. Such comparisons have been carried out via Welch’s *t* tests between the distributions of CF fluorescence values at *t* = 3 min for each enantiomer pair and are shown with corresponding violin plots next to each time-course graph **(C, F, I,** **L**). ****: *p*-value < 0.0001, **: *p*-value < 0.01. Numerical values of CF fluorescence in individual vesicles for each lipid type during the delivery of each substrate are provided in S1 and S2 Data.

To explore the possibility that an archaeal or hybrid membrane imposes permeability selection on different enantiomers, we compared permeability of four pentose sugars: deoxyribose, ribose, xylose, and arabinose (Fig 1). Both membranes demonstrate permeability to ribose, xylose, and arabinose and, importantly, show increased permeability to d-ribose compared to l-ribose (*P* < 0.01). Stereochemical selection was not evident for xylose or arabinose.

As discussed above, permeability for d-ribose has been reported for both fatty acid membranes and some eukaryotic-like phospholipids, but l- and d-permeabilities were not compared as part of these experiments [47]. These experiments did include a comparison of permeability for l- and d-xylose, demonstrating: (i) reduced permeability for both enantiomers of xylose compared to d-ribose and (ii) no difference in permeability between the two xylose enantiomers through POPC eukaryotic-like phospholipids [47], similar to our results for xylose in both the membranes tested here (Fig 1). Our experiments therefore also produce data consistent with the idea that d-ribose was favored over l- or d-xylose or d-arabinose by membrane selection (Figs 1 and S1).

l-deoxyribose showed very low average permeability through either membrane type but, in contrast, d-deoxyribose showed significantly increased permeability through both membrane types (*P* < 0.01 and *P* < 0.0001 for the archaeal and hybrid membrane, respectively). These results demonstrate that both the archaeal and hybrid membrane can drive stereochemical selection generating a ‘passive sorting mechanism’ [47] for d-ribose and d-deoxyribose. Equivalent comparisons of bacterial membrane mimics with diester bonds and fatty acid chains do not show stereochemical selection (S1 Fig). The patterns of stereochemical selection observed through the archaeal-like and hybrid membranes are consistent with the utilization of these compounds as the chemical building blocks for DNA and RNA, the molecules central to the hereditary, transcription, and translational systems; the core anabolic processes of all known life [82,83].

### Hexose sugar permeability through archaeal and hybrid membranes

As discussed in the introduction, another common function that likely emerged early in the evolution of life is the glycolysis pathway [19,20,67]. This pathway provides intermediate chemical precursors for both RNA and phospholipid compounds, suggesting a varied role in the evolution of life [68]. Although there is considerable differentiation in the glycolysis pathways across the tree of life, and in many cases enzymes of the pathway are not encoded by homologous genes [16,17], both archaea and bacteria can use glucose and fructose as a carbon source [84–86].

**Fig 2:**
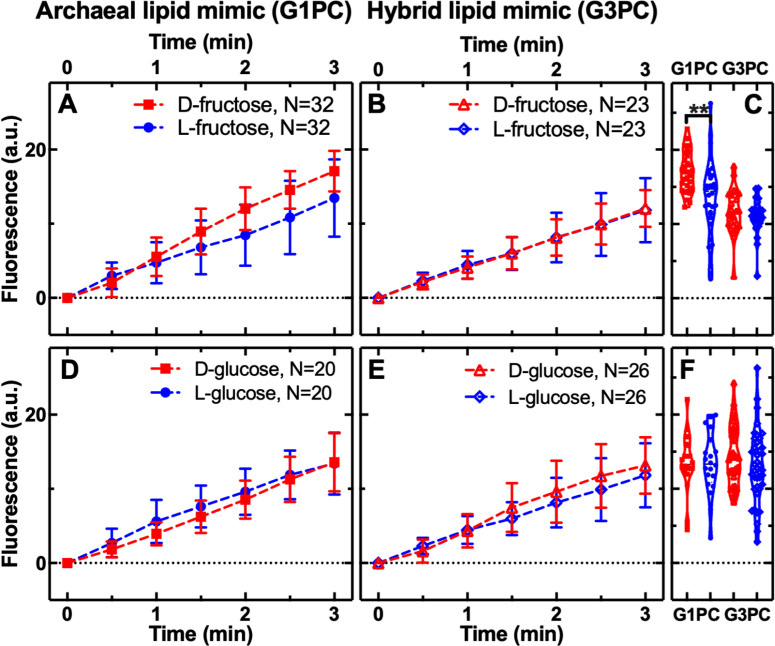
Hexose sugar permeability through archaeal and hybrid membranes. Temporal dependence of average carboxyfluorescein (CF) fluorescence in the archaea-like G1PC phospholipid membrane (**A**, **D**: G1PC, filled symbols) and in the ‘hybrid’ phospholipid membrane (**B, E**: G3PC, open symbols) during exposure to 1 mM of d- or l-fructose or glucose delivered to the microfluidic coves. Mean (symbols) and standard deviation (error bars) were calculated from at least 10 single-vesicle measurements across three independent experiments. Data are processed and illustrated as described in Fig 1 legend with statistical comparisons presented in (**C,** **F**). *N* is the number of single vesicles investigated for each substrate exposure and each type of vesicle. **: *p*-value < 0.01. Numerical values of CF fluorescence in individual vesicles for each lipid type during the delivery of each substrate are provided in S1 and S2 Data.

To understand how early cellular forms could make use of abiotically derived glucose or fructose [20,21] (both with four stereocenters), we performed permeability selection assays through the archaeal and hybrid membranes. These data show both hexoses can permeate through these membrane forms; but a minor selection for d-fructose over l-fructose enantiomers was observed through the archaeal-like diether isoprenoid membrane (*P* < 0.01), and no selection through the hybrid membrane (Fig 2). We also found no selection for l- or d-fructose enantiomers through a bacterial-like diester fatty acid membrane (S1 Fig). These data suggest utilization of an archaeal-like membrane could have generated a bias for d-fructose and therefore an enantiomeric metabolite bias for glycolysis-associated catabolism.

### Amino acid permeability through archaeal and hybrid membranes

**Fig 3:**
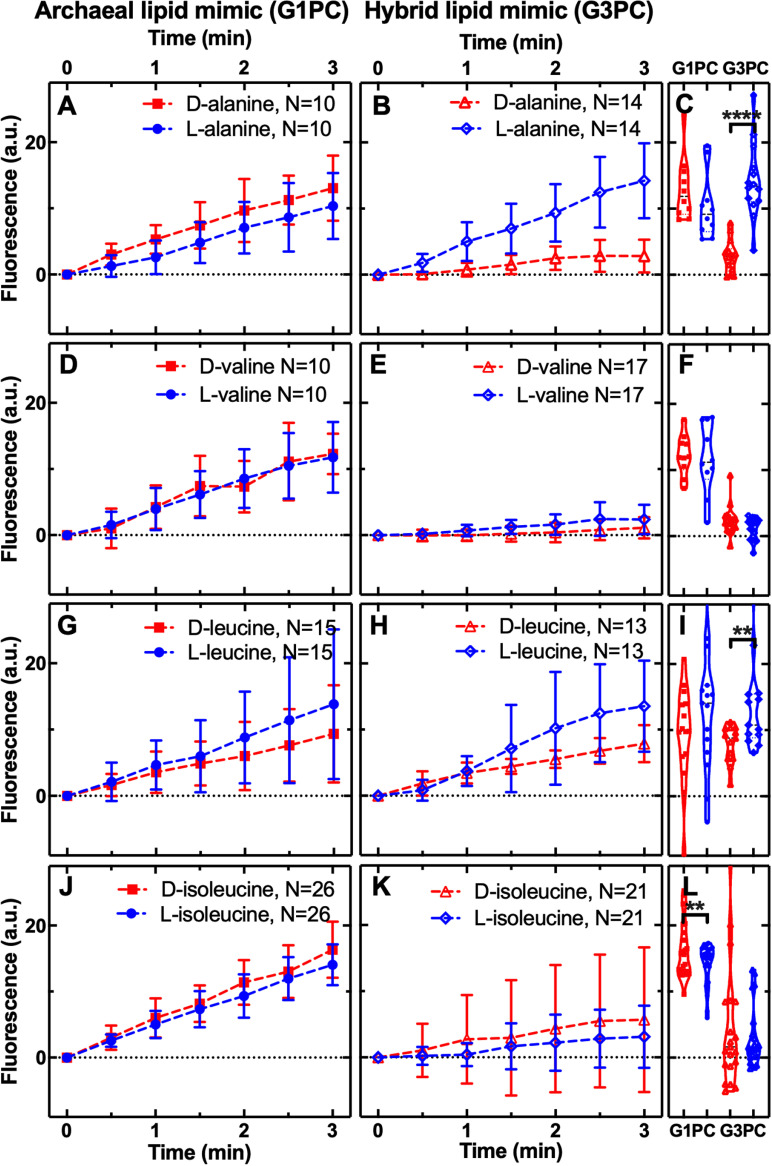
Archaeal and hybrid membrane permeability to non-polar amino acids. Temporal dependence of average carboxyfluorescein (CF) fluorescence in the archaea-like G1PC phospholipid membrane (**A, D, G, J**: G1PC, filled symbols) and in the ‘hybrid’ phospholipid membrane (**B, E, H, K**: G3PC, open symbols) during exposure to 1 mM of d- or l-alanine, valine, leucine, or isoleucine delivered to the microfluidic coves. Data are processed and illustrated as described in Fig 1 legend with comparisons reported in (**C, F, I, L**). Mean (symbols) and standard deviation (error bars) were calculated from at least 10 single-vesicle measurements across three independent experiments. *N* is the number of single vesicles investigated for each substrate exposure and each type of vesicle. ****: *p*-value < 0.0001, **: *p*-value < 0.01. Numerical values of CF fluorescence in individual vesicles for each lipid type during the delivery of each substrate are provided in S1 and S2 Data.

We previously demonstrated that archaeal-like diether isoprenoid membranes have a higher permeability to glycine and alanine compared to diester fatty acid bacterial membranes [79]. To explore if the archaeal and the hybrid membranes impose selection on alanine, we compared permeability of l- and d-alanine enantiomers. We found no differential selection through the archaeal membrane, but strong selection for the l-alanine enantiomer at 3-min exposure, in comparison to the effective exclusion of d-alanine in the hybrid membrane (*P* < 0.0001, Fig 3). We also note that we had previously demonstrated that l-alanine does not permeate through bacterial membranes with diester bonds and fatty acid chains [79]. This result demonstrates a statistically significant l-alanine sorting mechanism with l-alanine permeability 5-fold higher than d-alanine. Previous experiments, using a different approach and using DOPC/POPC membranes, had demonstrated a 1.2-fold permeability selection for l-alanine over d-alanine [45]. The alanine data are consistent with the amino acid stereochemical utilization of life, specifically in the hybrid G3P diether isoprenoid membrane and not the archaeal-like G1P diether isoprenoid membrane mimics tested here.

**Fig 4:**
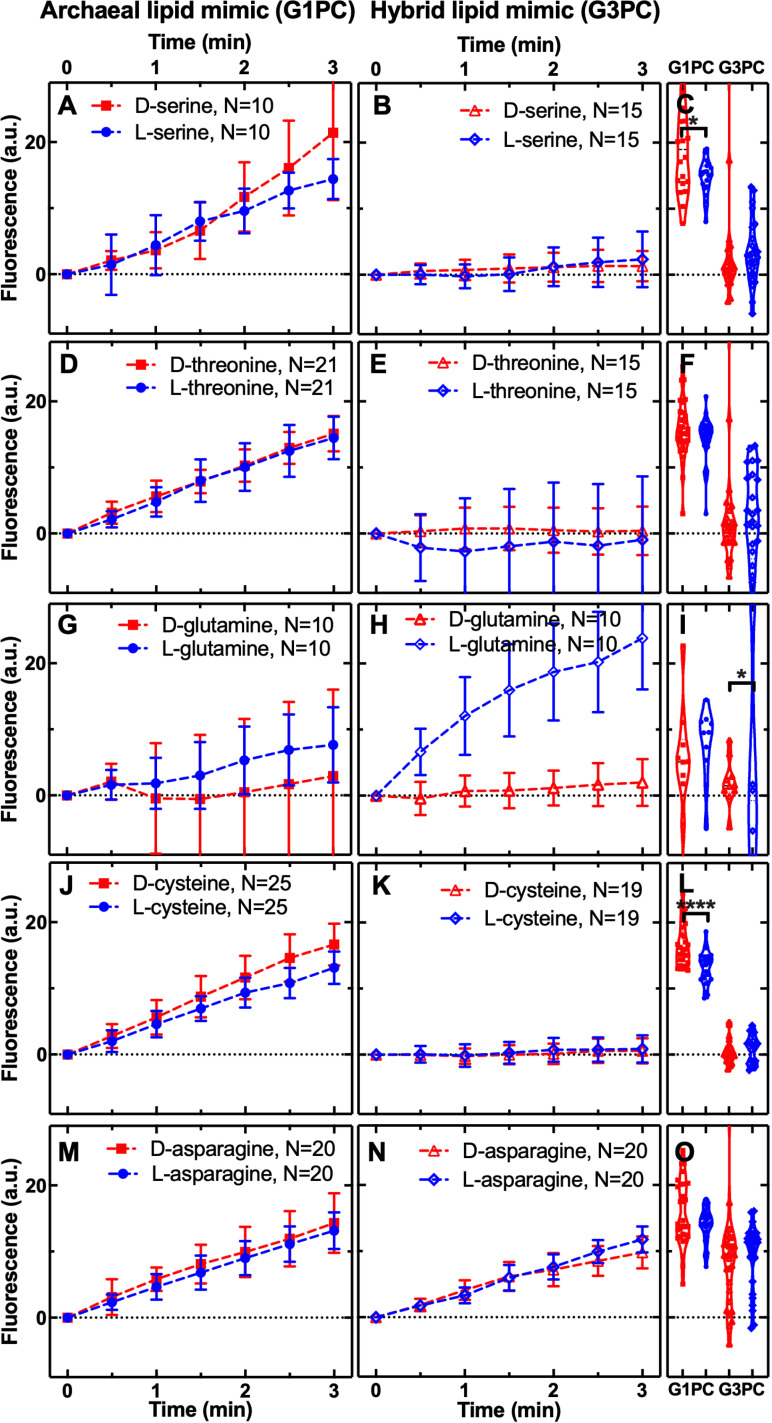
Polar amino acid permeability through archaeal and hybrid membranes. Temporal dependence of average carboxyfluorescein (CF) fluorescence in the archaea-like G1PC phospholipid membrane (**A, D, G, J, M**: G1PC, filled symbols) and in the ‘hybrid’ phospholipid membrane (**B, E, H, K, N**: G3PC, open symbols) during exposure to 1 mM of d- or l-serine, threonine, glutamine, or cysteine or asparagine delivered to the microfluidic coves. Data are processed and illustrated as described in Fig 1 legend with statistical comparisons presented in (**C, F, I, L, O**). Mean (symbols) and standard deviation (error bars) were calculated from at least 10 single-vesicle measurements across three independent experiments. *N* is the number of single vesicles investigated for each substrate exposure and each type of vesicle. ****: *p*-value < 0.0001, *: *p*-value < 0.05. Numerical values of CF fluorescence in individual vesicles for each lipid type during the delivery of each substrate are provided in S1 and S2 Data.

**Fig 5:**
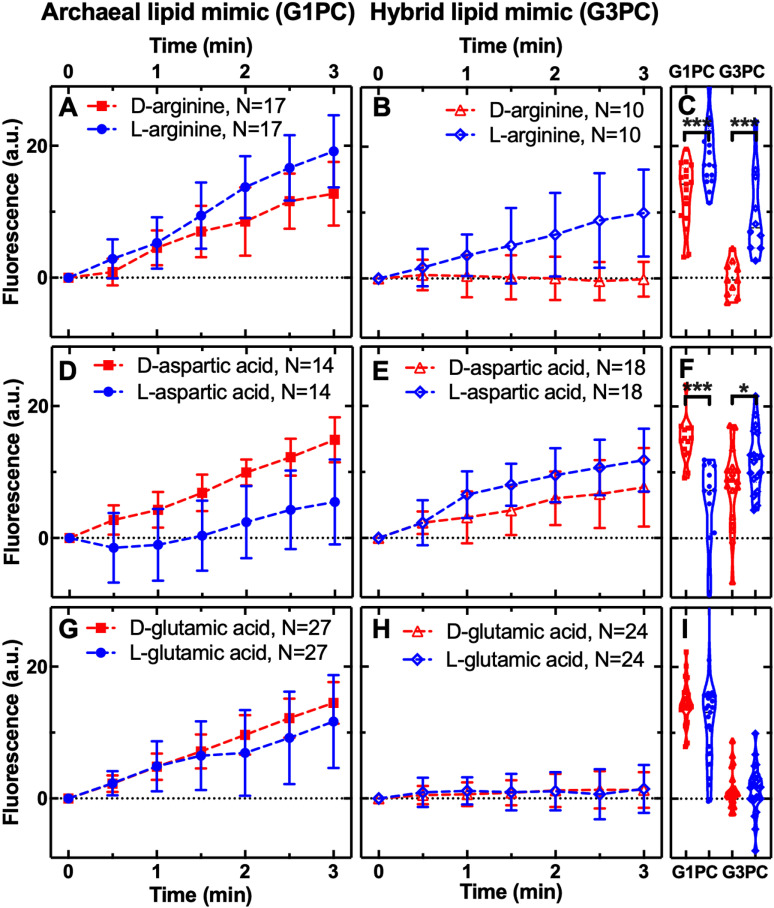
Charged amino acid permeability through archaeal and hybrid membranes. Temporal dependence of average carboxyfluorescein (CF) fluorescence in the archaea-like G1PC phospholipid membrane (**A, D, G**: G1PC, filled symbols) and in the ‘hybrid’ phospholipid membrane (**B, E, H**: G3PC, open symbols) during exposure to 1 mM of d- or l-arginine, aspartic acid, or glutamic acid delivered to the microfluidic coves. Data are processed and illustrated as described in Fig 1 legend with statistical comparisons presented in (**C, F, I**). Mean (symbols) and standard deviation (error bars) were calculated from at least 10 single-vesicle measurements across three independent experiments. *N* is the number of single vesicles investigated for each substrate exposure and each type of vesicle. ***: *p*-value < 0.001, *: *p*-value < 0.05. Numerical values of CF fluorescence in individual vesicles for each lipid type during the delivery of each substrate are provided in S1 and S2 Data.

To further explore stereochemical permeability selection, we performed permeability assays on 11 additional proteinogenic amino acids. These 11 amino acids included seven amino acids (V, D, E, S, L, T, I) that were produced in the Miller experiments, but at much lower molar concentrations than alanine or glycine [22,23]. The amino acids analyzed include compounds with two stereocenters (T, I) and a range of differing physical-chemical properties including: three non-polar (V, L, I, Fig 3), five polar (S, T, N, C, Q, Fig 4), one positively, and two negatively charged amino acids (R, D, E, respectively, Fig 5). These assays confirmed that all 14 amino acids tested (when including glycine and tryptophan previously analyzed [79]) permeated through the archaeal diether isoprenoid membrane (Figs 3–5). Furthermore, focusing on amino acids where enantiomers were compared: I, S, C, R, and D amino acids demonstrated enantiomer selection through the archaeal membrane. However, these results were mixed, with only arginine (R) showing l-selection while the other four (D, C, S, I) showed d-selection (Figs 3–5). This pattern is not consistent with how life evolved to use amino acid enantiomers.

**Fig 6:**
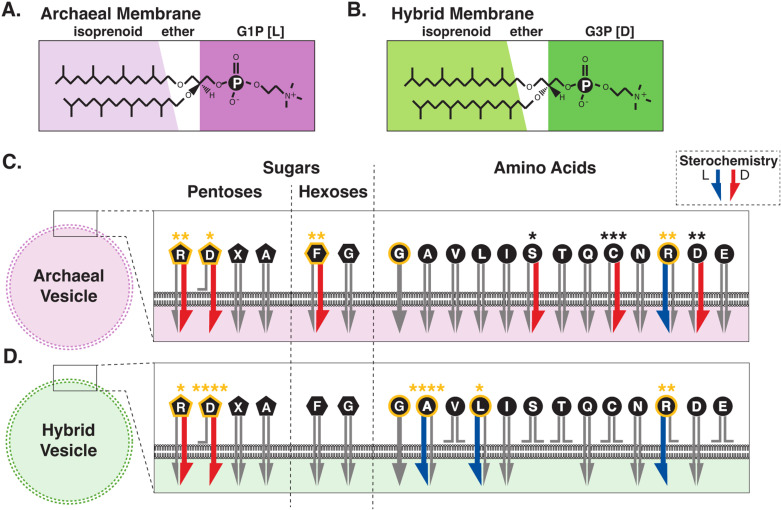
Summary of permeability selection results for the archaeal and hybrid membranes. (**A** and **B**) show the lipid compounds used for vesicle construction and permeability comparisons. (**C** and **D**) summarize permeability function. Pentose sugars are notated as pentagons, hexose sugars as hexagons, and amino acids as circles. Arrows indicate permeability function across the various membranes where wider blue (l) or red (d) arrows denote a significant differential enantiomer permeability selection. Benjamini–Hochberg corrected statistical significance are shown using the star convention (*P < 0.05, **P < 0.01, ***P < 0.001, and ****P < 0.0001), see Table 1. Key indicates which arrow side represents which enantiomer. Compounds colored orange indicate enantiomer selection that matches the core biochemistry of life. We have added glycine here based on results from our previous work. Glycine has no stereochemistry and has been shown to permeate both the archaeal and hybrid membrane [79].

**Table 1:** Summary of permeability and enantiomer selection data for the 18 substrates tested against the three membrane types.

Compounds tested	d-Archaeal permeability l/d	l- Hybrid permeability l/d	Archaeal enantiomer selection *P*-values	Hybrid enantiomer selection *P*-values	Bacterial membrane (*P*-value)
**Ribose**	**++/++++**	**+++/+++++**	0.0064**	0.0358*	**No selection (0.9124)**
**Deoxyribose**	**+/++++**	**+/+++++**	0.0177*	<0.0001****	**No selection (0.2716)**
**Xylose**	**+++/+++**	**+++/+++**	**0.8109**	**0.99**	**–**
**Arabinose**	**+++/+++**	**++/+++**	**0.3784**	**0.4141**	**No selection (0.6515)**
**Fructose**	**+++/++++**	**+++/+++**	0.0064**	**0.5303**	**No selection (0.6515)**
**Glucose**	**+++/+++**	**+++/+++**	**0.9214**	**0.301**	**–**
**Alanine (A)**	**+++/+++**	**+++/+**	**0.3969**	<0.0001****	**–**
**Valine (V)**	**+++/+++**	**+/+**	**0.9053**	**0.1771**	**–**
**Leucine (L)**	**+++/++**	**+++/++**	**0.3763**	0.0358*	**No selection (0.3276)**
**Isoleucine (I)**	**+++/++++**	**+/++**	**0.086**	**0.4893**	**–**
**Serine (S)**	**+++/+++++**	**+/+**	0.0358*	**0.8949**	**–**
**Threonine (T)**	**+++/++++**	**−/+**	**0.3714**	**0.5965**	**–**
**Glutamine (Q)**	**++/+**	**+++++/+**	**0.4761**	**0.0662**	**–**
**Cysteine (C)**	**+++/++++**	**+/+**	0.0009***	**0.8109**	**No selection (0.8122)**
**Asparagine (N)**	**+++/+++**	**+++/++**	**0.215**	**0.9124**	**–**
**Arginine (R)**	**++++/+++**	**++/−**	0.0064**	0.0064**	**No selection (0.3739)**
**Aspartic acid (D)**	**++/+++**	**+++/++**	0.0011**	**0.086**	**–**
**Glutamic acid (E)**	**+++/+++**	**+/+**	**0.1612**	**0.9124**	**–**

Carboxyfluorescein (CF) fluorescence after 3 min of exposure to each substrate at a concentration of 1 mM is summarized as follows: <0 = **−**; 0–5 a.u. = +, 5–10 a.u. = ++, 10–15 a.u. = +++, 15–20 a.u. = ++++, >20 a.u. = +++++. Statistical support for different enantiomer selection is indicated: ****: *p*-value < 0.0001, ***: *p*-value < 0.001, **: *p*-value < 0.01, *: *p*-value < 0.05, *P*-values are adjusted from Figs 1–5 to control for false discovery rates using Benjamini–Hochberg, 1995 correction [87]. Enantiomer selection consistent with the stereochemical bias of life is colored blue. Enantiomer selection that contradicts the stereochemical bias of life is colored orange. Absence of enantiomer selection, where the same compound is not used for core anabolic metabolism of life, is colored green.

In contrast, stereochemical permeability assays through the hybrid membrane (a phospholipid which has the opposite (d-) stereochemistry to the archaeal membrane [58]) demonstrated fewer amino acids could permeate this membrane. Specifically, C, E, S, T, and V showed no permeability, in contrast to the archaeal membrane (Figs 3–5). Consistent with the alanine results discussed above, the hybrid membrane showed l-enantiomer selection for L, Q, R, and D amino acids (Figs 3–5), demonstrating how this membrane can generate passive selection for a reduced alphabet of l-amino acids. These results show that specific chemical characteristics of the membrane phospholipid can determine membrane permeability function. We also note, as the hybrid membrane effectively excluded multiple amino acid enantiomers, effectively reducing enantiomer pollution that could curtail abiotic amino acid polymerization, these experiments confirm that the amino acid selectivity represents true differences in membrane permeability function (see also S3 Fig for further controls).

To test for enantiomer selection through a bacterial model membrane we compared l- and d-permeability of R, C, and L amino acids through a bacterial mimic with a G3P head group with diester links and a fatty acid tail. These amino acids were chosen as they showed very different enantiomer permeability through archaeal-like and hybrid membranes (Figs 3–6), and C and L amino acids were previously tested against eukaryotic like DPPC membranes [44]. Our experiments demonstrate no evidence of enantiomer selection through the bacterial-like G3PE/G3PG membrane (S2 Fig) for R, C, and L amino acids. While our results for l-leucine showed no significant difference in permeability for the G3PE/G3PG membranes, this is in contrast to previous results for the eukaryotic-like DPPC membrane, which suggest a slight increased permeability for l-leucine [44]. This study also demonstrated that the d-DPPC membrane showed no permeability for either l- or d-cysteine. We note however the DPPC membrane experiments were conducted over 50 h demonstrating an effect, where present, at 15 h [44] while the experiments and effect reported here were identified over 3 min.

The amino acid results are intriguing; the difference between the archaeal-like and hybrid diether isoprenoid membranes is the presence of a G1P versus G3P head group, a chemical change that reverses the chirality of the membrane (l- to d-). Interestingly, this change drastically alters the permeability selection for a subset of amino acids, for example, switching enantiomer selection for aspartic acid from d- to L-, maintaining l-arginine selection in both membrane types (a similar pattern to that seen for both deoxyribose and ribose), while changing permeability of both enantiomers across the archaeal membrane to relative exclusion across the hybrid membrane for valine (V), glutamic acid (E), serine (S), threonine (T), and cysteine (C). These data suggest that the chemical characteristics of substrate-membrane interaction do operate like a lock and key mechanism [44].

### Strong permeability selection matches the core metabolic network of life

The Benjamini–Hochberg [87,88] corrected *P*-values for all the experiments reported above are shown in Table 1 and Fig 6 and confirm that both membrane types show permeability for only a subset of the metabolites tested. The types of metabolites that permeate, and the enantiomer selection, varies between the two membranes, a pattern of characteristics expected from a lock and key model [44]. Indeed, the observation that only a subset of compounds permeate each membrane, and that this subset changes based on changes in the membrane chemistry, is consistent with the proposal that membranes can act as substrate recognition structures [44]. However, both membrane types show strong selection for d-ribose and d-deoxyribose. Furthermore, the hybrid diether isoprenoid membrane only shows enantiomer selection for a subset of amino acid l-enantiomers excluding many of the d-forms tested. Strikingly, the most potent l-amino acid selection was seen for alanine (Table 1 and Fig 6). We therefore argue that the important conclusion is not that the experiments reported here show a membrane which has wide ranging enantiomer selection, which would make such a membrane leaky rather than selective. Instead, our main conclusion is that the selection is strong and specific for a defined subset of compounds that match early core anabolic metabolic requirements: d-ribose, d-deoxyribose, and l-alanine; a pattern that also acts to minimize enantiomer pollution.

## Discussion

Using controlled unilamellar vesicle experiments, we demonstrate that a model archaeal membrane and one hybrid membrane-form derive stereochemical permeability selection (summarized in Table 1 and Fig 6). These data suggest an ancient phase of evolution based on either of the two membrane types, or a membrane with a similar selective function, could have driven selection for the stereochemical characteristics of proto-metabolism. Specifically, an evolutionary bottleneck, dependent on either form of diether isoprenoid phospholipid membrane chemistry tested here, would have generated selection for d-ribose and d-deoxyribose, hard-wiring the use of right-handed ribonucleotides. Strikingly, a cell type with a hybrid G3P diether isoprenoid membrane—a possible precursor of both archaeal and bacterial membrane types—could have hardwired both the use of d-ribonucleotide oligonucleotides and l-amino acids, although initially with a reduced alphabet of amino acids. We note that this selection would have been reinforced by amino acid polymerization, as biochemical pathways for the production of amino acids produce achiral precursors, meaning chiral selection could not have been passed through known extant metabolic networks. However, the d-stereochemical selection demonstrated here for ribose could carry through to RNA, where it could determine amino acid l-stereochemistry through preferential transfer of l-aminoacyl residues to tRNA (e.g., [7–9]). Furthermore, recent work has demonstrated that a single candidate ancestral reaction can abiotically generate all the phosphorylation reactions needed in core metabolism [15] including the production of ribose-5-phosphate which, if located within membranes with a similar permeability selection function as demonstrated here, would be one step closer to the formation of d-ribonucleic acids.

Given current data, we advocate for a phase of evolution prior to, or at, the last universal common ancestor, which was a hybrid membrane consisting of diether lipids with isoprenoid chains containing methyl branches (archaeal traits) bonded to a G3P with right-handed stereochemistry (bacterial traits) or an alternative membrane with similar permeability selection characteristics. We note that this scenario represents only one phase of membrane evolution in a long history of possible membrane transitions between the first ‘life’ form and the last universal common ancestor. As such this scenario is compatible with many previously suggested ancestries for the prokaryotic domains (e.g., [34,68,69]). Phylogenomic analyses have suggested that the last universal last common ancestor lies between the Archaea and the Bacteria [70–75], or potentially within the Bacteria [76–78]. Our results do not directly inform this debate, as multiple transitions in membrane chemistry are possible; indeed, the transition through the hybrid membrane we advocate could have occurred before the derivation of either the archaeal or the bacterial domains of life.

Although there is evidence that phospholipids can form abiotically [60] and that precursors of phospholipids can form in synthetic early Earth environments [20], there is currently no evidence that the hybrid phospholipid forms tested here could have formed abiotically in high and pure concentrations during phases on the early Earth, although we note isoprenoid chains have been shown to enhance the stability of prebiotic membranes [89]. It is therefore important to acknowledge that a pure-form membrane, as used here, is implausible as an early proto-cellular membrane, especially if it was derived from an abiotic source [60] and occurred prior to the emergence of a dedicated metabolic network for the generation of membrane-forming compounds. Furthermore, it is not clear, at this stage, if mixed membranes encompassing the types of phospholipids used here (or alternatives with equivalent permeability function) are viable, although experiments with *Escherichia coli* suggest engineered variant lipids can form mixed membranes [64] while direct observations have suggested natural bacteria can possess membranes of mixed composition and stereochemistry [65,66]. Therefore, these data suggest that it is possible to form mixed phospholipid unilamellar vesicles. A key future question is therefore, could such membranes generate similar patterns of permeability selection identified in this paper. Future work should therefore focus on assessing the stability and permeability function of mixed membranes.

It is also important to acknowledge that the data presented here are silent on the evolution of both membrane biosynthesis and core metabolic biosynthetic pathways and the relative timing of each. This leads to a problem faced by many hypotheses regarding the origins of protocells; which came first: membrane compartmentalization or biosynthetic metabolism? Many scenarios suggest a catalytic RNA came first [30,53–57]. Hypothetically, if such a molecule were to relocate to the membranes studied here (or membranes with similar permeability functions), this molecule could self-replicate with reduced exposure to non-compatible ribose enantiomers; such membranes would therefore allow selection for chiral bias by excluding contaminations from racemic mixes (enantiomer pollution) ([1,3,11,12]). Consequently, enrichment of one ribose enantiomer within the membranes studied here would allow faster and stable replication and therefore Darwinian selection. Such a scenario could allow for two additional transitions: (i) adoption of systems such as DNA or amino acid polypeptides and then (ii) evolution of metabolic pathways. Again, if this evolutionary process was located within a membrane with similar permeability characteristics as shown here for the hybrid membrane, both DNA and amino acid systems could be adopted with highly reduced enantiomer pollution, allowing for polymerization to occur and the DNA–RNA and RNA–amino acid associations that underpins the central dogma to evolve. This process could then, in turn, allow for the evolution of protein catalysis and metabolic pathways. We note that in the hybrid membrane tested here, this membrane acted to heavily exclude both l-deoxyribose and many d-amino acids. This is just one scenario in which membrane permeability could have underpinned the evolution of core cellular systems. It is also conceivable that a biosynthetic metabolism, producing enantiomerically pure amino acids and sugars, emerged within a membrane that did not initially allow for enantiomeric selection (following on from the scenarios outlined in [2,5–9], for example). The subsequent emergence of a membrane with the permeability characteristics identified here would have then reinforced the chiral bias by excluding contaminations from racemic mixes which would disrupt polymerization thereby further selecting for chiral biosynthetic metabolism. We acknowledge both scenarios presented are hypothetical and that we cannot explain where, when and how a membrane with this type of permeability function arose abiotically.

The results reported here also demonstrate that additional bottlenecks, through an archaeal G1P diether isoprenoid membrane, could have hard-wired bias for d-hexose sugars for metabolic utilization such as glycolysis, as demonstrated by our result of increased archaeal membrane permeability to d-fructose (Fig 2). However, this was not a strong result, demonstrating low differential selection for d-fructose. Furthermore, the possibility of two bottlenecks with differing membrane chemistries leaves some confusion regarding the relative inferred order of membrane utilization relative to the tree of life. We also note that glycolytic catabolism of hexose sugars does not impose similar constraints on enantiomer utilization as would the emergence of the anabolic polymerization of ribonucleic acids and amino acid polypeptides [1]. This is because hexose compounds are primarily broken down for metabolic benefit and not polymerized for storage or translation of information in the form of DNA, RNA, and amino acid polypeptides. Differential utilization, i.e., anabolic polymerization in the case of amino acids and ribonucleotides versus catabolic metabolism in the case of glycolysis of hexoses, therefore suggests that the stereochemical permeability selection on precursors of glycolysis is not as important a factor as precursors of the compounds utilized in the central dogma. For these reasons, we suggest enantiomer membrane permeability selection for catabolic precursors of glycolysis was not an important factor compared to selection for subunits utilized in anabolic metabolism.

We acknowledge that wider analysis of membrane types, including additional bacterial- and eukaryotic-mimics may reveal additional permeability selection traits informative for understanding proto-cellular membrane evolution [44,45]. We have partly investigated this possibility in a bacterial-type diester fatty acid membrane mimic for three pentose sugars, one hexose sugar, and three amino acids (S1–S3 Figs). These were chosen as they showed a wide variation of permeability characteristics in the archaeal and the hybrid membranes tested (Table 1). In all these tests the bacterial membrane mimic demonstrated no stereochemical selection within the time frame of our experimental observations (3 min), an outcome that could be a function of their lower permeability, as identified in our previous experiments [79].

## Conclusions

In the Introduction, we discussed published examples of chemical polymerization of nucleotides and amino acids in abiotic conditions [1,3,11,12]. In these examples, both types of polymerization are limited when conducted in racemic mixes of substrate. The membrane permeability selection demonstrated here would solve this problem by providing a compartment with specific enantiomer enrichment, allowing polymerization to progress without the inhibition generated by racemic mixes of the constituent substrates. Such a system would have multiple advantages in competition over protocells without enantiomer selection. Specifically, both permeability and polymerization selection would act synergistically to generate competitive advantage and therefore Darwinian selection [40,41]. Understanding how life began is a ‘jigsaw puzzle’ [90]; the utilization of a hybrid G3P diether isoprenoid phospholipid membrane (or a membrane with similar permeability functions) to derive stereochemical selection for deoxyribose, ribose and a reduced alphabet of amino acids (Table 1 and Fig 6) is a potential piece of that puzzle. However, we acknowledge that we currently cannot explain how, when and where a pure membrane similar to the hybrid membrane tested here could have emerged. We therefore recognize the need to test for the phenomenon of chiral selection in a wide range of candidate membrane chemistries, including mixed membranes. Finally, we note that these data identify alternative membrane chemistries that are potentially useful for isolating enantiomeric forms, a challenge in the use of many abiotically produced pharmaceutical compounds [91,92].

## Methods

All methods are adaptations of those reported in Łapińska and colleagues 2023 [79] and at https://dx.doi.org/10.17504/protocols.io.rvvd666.

### Preparation of materials

All metabolites were purchased from Merck with chemical purity reported in S1 Table. All Lipids were purchased from Avanti Polar Lipids [i.e., 1,2-di-O-phytanyl-sn-glycero-1-phosphocholine, 1,2-di-O-phytanyl-sn-glycero-3-phosphocholine, 1,2-dioleoyl-sn-glycero-3-phosphoethanolamine, 1,2-dioleoyl-sn-glycero-3-phospho-(1′-rac-glycerol) and 1,3-bis(sn 3′-phosphatidyl)-sn-glycerol)]. Indium tin oxide (ITO)-coated glass slides were purchased from VisionTek Systems. The fluorescent probe 5(6)-carboxyfluorescein (CF), (MW = 376 g/mol), was dissolved in absolute ethanol at a stock concentration of 10 mg/mL. In order to perform all permeability experiments at physiological pH (7.4), the washing buffer was prepared by dissolving sucrose (MW = 342 g/mol) in 5 mM 4-(2-hydroxyethyl)-1-piperazineethanesul-fonic acid (HEPES) at pH = 7.4 at a final sucrose concentration of 195 mM. All metabolites were dissolved in the washing buffer to a final concentration of 1 mM.

### Preparation of synthetic vesicles

Giant unilamellar vesicles were electroformed using a Vesicle Prep Pro (Nanion) [93]. Three different lipid stocks; archaeal lipid mimic (G1PC) (2,3-di-O-phytanyl-sn-glycero-1-phosphocholine), hybrid lipid mimic (G3PC) (1,2-di-O-phytanyl-sn-glycero-3-phosphocholine) and bacterial lipid mimic mixed of three lipids: 1,2-dioleoyl-sn-glycero-3-phosphoethanolamine, 1,2-dioleoyl-sn-glycero-3-phospho-(1′-rac-glycerol) and 1,3-bis(sn-3′-phosphatidyl)-sn-glycerol were prepared to a final concentration of 10 mM dissolved in chloroform (S2 Table). Next, 10 µL of each lipid solution was spread evenly onto the conductive side of an ITO-glass slide and vacuum desiccated to dry the solution. Meanwhile, 495 µL of washing buffer was degassed and mixed with 5 µL of 10 mg/ml of 5(6)-CF solution to a final concentration of 0.266 mM. The lipid-coated ITO-glass slide was loaded into the Nanion and a greased rubber O-ring was placed over the dried layer. The area inside the O-ring was filled with 300 µL of 0.266 mM CF solution and another ITO-glass slide conductive side down was placed over, avoiding the formation of bubbles. Different electroformation protocols were implemented depending on the lipid used (see S3 Table), all preparations were carried out at 37 °C. The final suspension contains CF fluorescent vesicles and free CF molecules, which were stored at 4 °C for 2 days maximum.

### Fabrication of the microfluidic device

The microfluidic device is described in detail in Łapińska (2023) [79]. In short, the device contains two inlets connecting to a main chamber split into four channels; these contain hydrodynamic traps (also known as coves) with the chambers finally leading to a single outlet. Using a mold created by multilevel photolithography [94], polydimethylsiloxane (PDMS) replicas of the device were produced. In summary, a 10:1 (base:curing agent) PDMS mixture (SYLGARD 184 Silicone Elastomer Kit, Dow) was poured over the mold and degassed for 30 min, this was cured for 2 h at 70 °C in an oven. The PDMS was peeled from the mold and a 1.5 mm biopsy punch was used to create fluidic access. To irreversibly seal the PDMS replica to a glass slide, oxygen plasma treatment was used (10-s exposure to 30W plasma power, Plasma etcher, Diener Electronic GmbH).

### Microfluidic permeability assay

Washing buffer was injected into the buffer inlet until the device was flooded completely and escaping the metabolite inlet and outlet. The metabolite inlet was then sealed with tape and 10 µL of the fluorescent vesicle suspension was pipetted into the washing inlet. The tape prevented fluorescent vesicle suspension entering the metabolite inlet. The chip was mounted onto an inverted epifluorescence microscope (Olympus IX73) equipped with a sCMOS camera (Zyla 4.2, Andor, used at an exposure time of 0.1s), a blue LED (CoolLED pE300white, used at 30% of its intensity) and a FITC filter. Two pre-prepared 1 mL syringes were filled with; i) washing buffer, ii) 1 mM of a compound of interest and were connected to 23-gauge needles (Becton Dickinson) and then Masterflex Transfer Tygon tubing with 0.5 mm inner and 1.5 mm outer diameter (Cole-Parmer Instrument). These were controlled by a Nemesys pump operated via the Qmix Elements software (Centoni). The tape was removed from the metabolite inlet and the tubing containing the metabolite of interest, was inserted flowing at a rate of 0.5 µL/h. Then, the tubing containing the washing buffer was inserted into the remaining inlet, flowing at a rate of 5 µL/h. These flow rates were increased in a stepwise fashion at steps of 0.5 µL/h up to 2 µL/h for the metabolite solution and 5 µL/h up to 25 µL/h for the washing buffer. These were kept constant for 20 min to remove any free CF molecules from the microfluidic device. After this time the metabolite solution was increased to 25 µL/h and the washing buffer solution was reduced to 1 µL/h. Brightfield and fluorescent imaging of an area containing 14 coves were acquired every 30 s for 3 min. All measurements were carried out at room temperature (i.e., 22 °C).

### Vesicle-free carboxyfluorescein and metabolite interaction assays

To explore the possibility that compound stereochemical variation altered carboxyfluorescein function, we ran separate microplate reader experiments demonstrating that the stereochemistry of the metabolic compound does not have a differential effect on the fluorescence of carboxyfluorescein (S3 Fig), the reporter used throughout these experiments. These data provide further control for our permeability selection data reported above.

Washing buffer was used to dissolve CF to a concentration of 0.532 mM and 100 µL was added to wells of a 96-well plate (Costar 96*,* Fisher Scientific). Then, 100 µL of 1 mM metabolite solution was added to each well and the plate placed within a Clariostar Plus plate reader (BMG Labtech, UK) for fluorescence data acquisition over 30 min (Excitation 483-14, Emission 596, with a FITC filter). Three independent experiments were carried out for each metabolite.

### Image and data analysis

Images for each membrane/metabolite combination tested, at every time point, were exported into ImageJ for quantitative comparative analysis. Only single unilamellar vesicles were retained for analysis and a circle was drawn around each vesicle. A second identical circle was drawn in an area 10 µm upstream of the vesicle, which was then used to produce subtracted values for intra-vesicle fluorescence. The initial intra-vesicle fluorescence value (*t* = 0) was subtracted. The values were then corrected for the impact of both the delivery of the washing buffer solution and photobleaching on the intra-vesicle CF fluorescence signal (see Methods detailed in [95]). The permeability of each membrane mimic was measured in three independent experiments. Statistical comparisons were carried out using Welch’s *t* test and multiple comparisons were conducted using the Benjamini–Hochberg method in R v 4.3.2 [96]. The data analyses presented encompass 43 *t* tests (Figs 1–5 and S1–S3). Although each *t* test represents an a priori hypothesis test rather than a data sift, it is prudent in such situations to use a correction for false discovery rates, namely the Benjamini–Hochberg, 1995 correction [87,88]; the results of these statistical corrections are shown in Table 1.

## Supporting information

S1 Fig Sugar enantiomer permeability through bacterial membranes. Temporal dependence of average carboxyfluorescein (CF) fluorescence in the bacteria-like phospholipid membrane during the exposure to 1 mM of ribose (**A**), deoxyribose (**C**), arabinose (**E**) or fructose (**G**) delivered to the microfluidic coves. Mean (symbols) and standard deviation (error bars) were calculated from at least 10 single-vesicle measurements across three independent experiments. Lines are guides for the eye. *N* is the number of single vesicles investigated for each substrate exposure. *N* varies across different substrate experiments investigated due to technical constraints. However, care has been taken to obtain the same *N* for each substrate experiment across the two different enantiomer treatments in order to ensure reliable statistical comparisons. Such comparisons have been carried out via Welch’s *t* tests between the distributions of CF fluorescence values at *t* = 3 min for each enantiomer and are shown with corresponding violin plots next to each time-course graph **(B, D, F, H)**. ****: *p*-value < 0.0001, **: *p*-value < 0.01. Numerical values of CF fluorescence in individual vesicles during the delivery of each substrate are provided in S3 Data.
(PDF)

S2 Fig Amino acid enantiomer permeability through bacterial membranes. Temporal dependence of average carboxyfluorescein (CF) fluorescence in the bacteria-like phospholipid membrane during the exposure to 1 mM of variant cysteine (**A**), arginine (**C**), and leucine (**E**) delivered to the microfluidic coves. Mean (symbols) and standard deviation (error bars) were calculated from at least 10 single-vesicle measurements across three independent experiments. Lines are guides for the eye. *N* is the number of single vesicles investigated for each substrate exposure. *N* varies across different substrate experiments investigated due to technical constraints. However, care has been taken to obtain the same *N* for each substrate experiment across the two different enantiomers in order to ensure reliable statistical comparisons. Such comparisons have been carried out via Welch’s *t* tests between the distributions of CF fluorescence values at *t* = 3 min for each enantiomer and are shown with corresponding violin plots next to each time-course graph **(B, D, F)**. ****: *p*-value < 0.0001, **: *p*-value < 0.01. Numerical values of CF fluorescence in individual vesicles during the delivery of each substrate are provided in S3 Data.
(PDF)

S3 Fig Metabolite stereochemistry does not have an impact on carboxyfluorescein fluorescence in solution. Temporal dependence of **(A)** carboxyfluorescein (CF) fluorescence alone or in the presence of d- or l-enantiomers (red squares or blue circles, respectively) of **(B–E)** sugars, or **(F–J)** amino acids. Mean (symbols) and standard deviation (error bars) were calculated from three independent experiments performed in 96-well plates with fluorescence measured via a plate reader (note the reduced scale of the Y axis compared to the other plots reported). 100 µL of 0.532 mM CF was added to 100 µL of 1 mM metabolite; these were the concentrations used in the permeability experiments above. Numerical values of CF fluorescence in individual experiments in the presence of each metabolite are provided in S4 Data.
(PDF)

S1 Table Chemical purity of the metabolites investigated. (DOCX)

S2 Table Details of membrane phospholipid compounds used for vesicle synthesis. (DOCX)

S3 Table Parameters used for electroformation of vesicles. (DOCX)

S1 Data Archaeal lipid mimic. Data for permeability assays across the archaeal-like phospholipid mimic.
(XLSX)

S2 Data Hybrid lipid mimic. Data for permeability assays across the hybrid phospholipid mimic.
(XLSX)

S3 Data Bacterial lipid mimic. Data for permeability assays across the bacterial-like phospholipid mimic.
(XLSX)

S4 Data Data for control experiment to assess impact of ‘stereochemistry impact on dye’. (XLSX)

S5 Data R code for Benjamini–Hochberg corrected *P*-value calculations. (R)

S6 Data *P*-value table. (CSV)

## Abbreviations

DMPC, 1,2-dimyristoyl-sn-glycero-3-phosphocholine; DOPC 1,2-dioleoyl-sn-glycero-3-phosphocholine; DPPC, 1,2-dipalmitoyl-sn-glycero-3-phosphocholine; EPC, egg yolk phosphatidylcholine; G1P, glycerol-1-phosphate; G1PC, 2,3-di-O-phytanyl-sn-glycero-1-phosphocholine; G3P, glycerol-3-phosphate; G3PC, 1,2-di-O-phytanyl-sn-glycero-3-phosphocholine; ITO, indium tin oxide; LUCA, last universal common ancestor; PDMS, polydimethylsiloxane; POPC, 1-palmitoyl-2-oleoyl-sn-glycero-3-phosphocholine; tRNA, transfer RNA.
