# Supplementary figures and images for: Permeability selection of biologically relevant membranes matches the stereochemistry of life on Earth
Source: PLoS Biol. 2025 May 20;23(5):e3003155. doi: 10.1371/journal.pbio.3003155 (PMC12091744; doi:10.1371/journal.pbio.3003155)

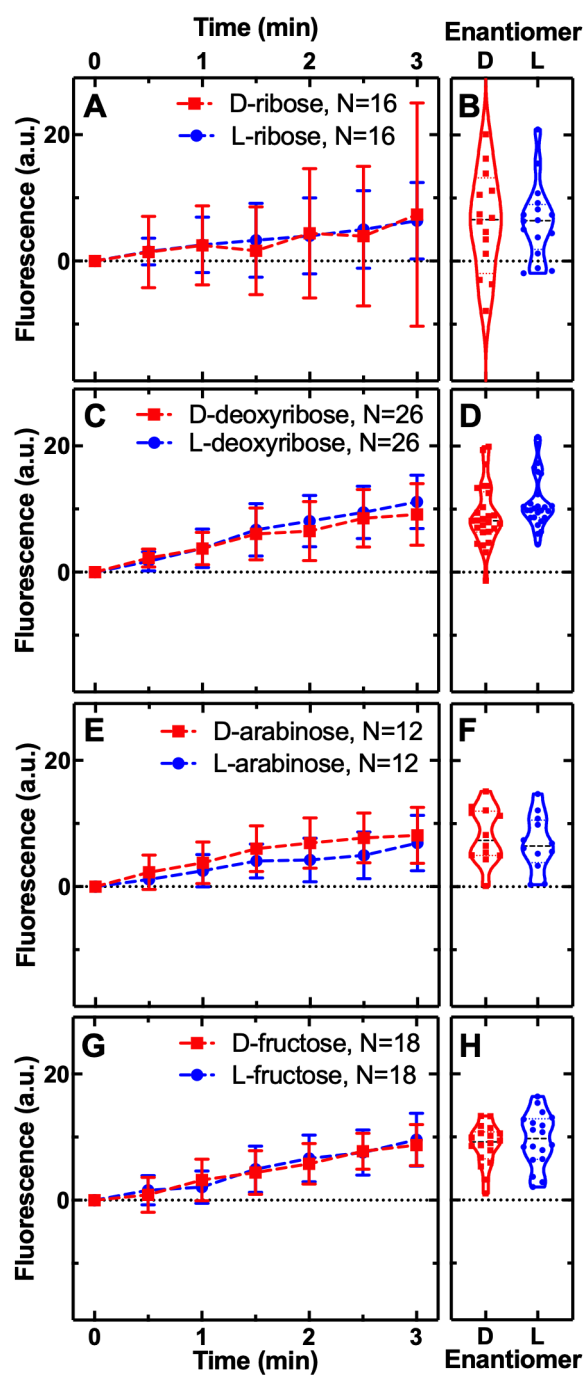

Supplement: S1 Fig — Temporal dependence of average carboxyfluorescein (CF) fluorescence in the bacteria-like phospholipid membrane during the exposure to 1 mM of ribose (A), deoxyribose (C), arabinose (E) or fructose (G) delivered to the microfluidic coves. Mean (symbols) and standard deviation (error bars) were calculated from at least 10 single-vesicle measurements across three independent experiments. Lines are guides for the eye. N is the number of single vesicles investigated for each substrate exposure. N varies across different substrate experiments investigated due to technical constraints. However, care has been taken to obtain the same N for each substrate experiment across the two different enantiomer treatments in order to ensure reliable statistical comparisons. Such comparisons have been carried out via Welch’s t tests between the distributions of CF fluorescence values at t = 3 min for each enantiomer and are shown with corresponding violin plots next to each time-course graph (B, D, F, H). ****: p-value < 0.0001, **: p-value < 0.01. Numerical values of CF fluorescence in individual vesicles during the delivery of each substrate are provided in S3 Data. (PDF) [file pbio.3003155.s001.pdf]

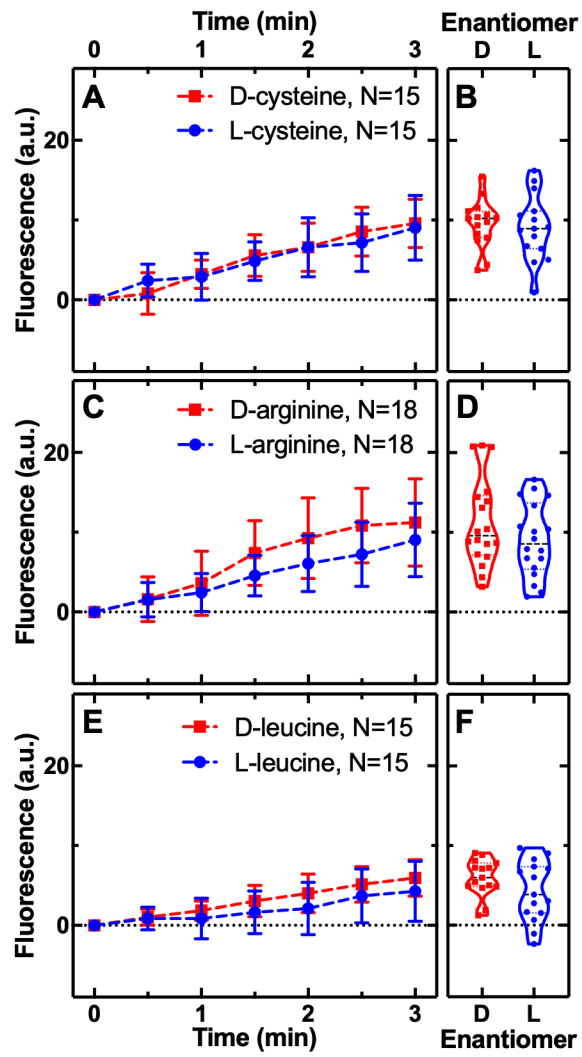

Supplement: S2 Fig — Temporal dependence of average carboxyfluorescein (CF) fluorescence in the bacteria-like phospholipid membrane during the exposure to 1 mM of variant cysteine (A), arginine (C), and leucine (E) delivered to the microfluidic coves. Mean (symbols) and standard deviation (error bars) were calculated from at least 10 single-vesicle measurements across three independent experiments. Lines are guides for the eye. N is the number of single vesicles investigated for each substrate exposure. N varies across different substrate experiments investigated due to technical constraints. However, care has been taken to obtain the same N for each substrate experiment across the two different enantiomers in order to ensure reliable statistical comparisons. Such comparisons have been carried out via Welch’s t tests between the distributions of CF fluorescence values at t = 3 min for each enantiomer and are shown with corresponding violin plots next to each time-course graph (B, D, F). ****: p-value < 0.0001, **: p-value < 0.01. Numerical values of CF fluorescence in individual vesicles during the delivery of each substrate are provided in S3 Data. (PDF) [file pbio.3003155.s002.pdf]

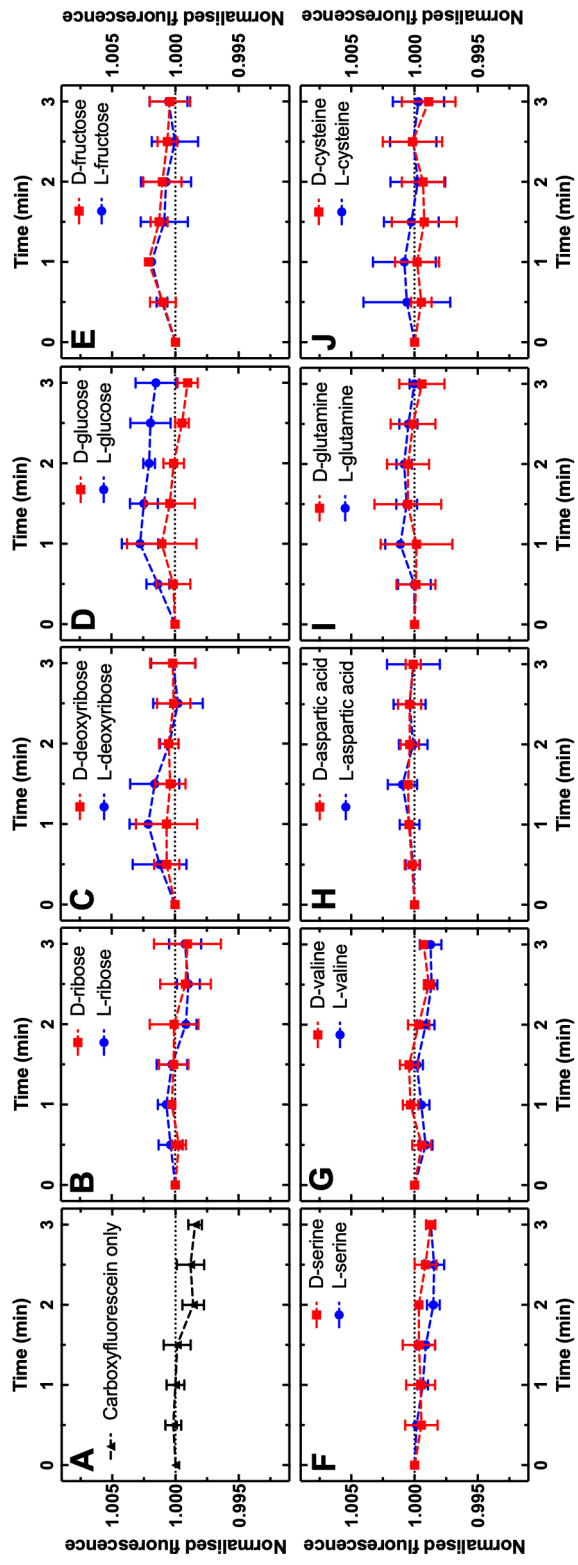

Supplement: S3 Fig — Temporal dependence of (A) carboxyfluorescein (CF) fluorescence alone or in the presence of d- or l-enantiomers (red squares or blue circles, respectively) of (B–E) sugars, or (F–J) amino acids. Mean (symbols) and standard deviation (error bars) were calculated from three independent experiments performed in 96-well plates with fluorescence measured via a plate reader (note the reduced scale of the Y axis compared to the other plots reported). 100 µL of 0.532 mM CF was added to 100 µL of 1 mM metabolite; these were the concentrations used in the permeability experiments above. Numerical values of CF fluorescence in individual experiments in the presence of each metabolite are provided in S4 Data. (PDF) [file pbio.3003155.s003.pdf]
