# Supplementary material for: Permeability selection of biologically relevant membranes matches the stereochemistry of life on Earth
Source: PLoS Biol. 2025 May 20;23(5):e3003155. doi: 10.1371/journal.pbio.3003155 (PMC12091744; doi:10.1371/journal.pbio.3003155)
Supplement: S1 Table — (DOCX) [file pbio.3003155.s004.docx]

**Table S1 Chemical purity of the metabolites investigated.**

| **Metabolite** | **Product number** | **Purity level** |
| --- | --- | --- |
| D-(-)-Ribose | R7500 | ≥99% |
| L-(+)-Ribose | [R4377](https://www.sigmaaldrich.com/GB/en/product/sigma/r4377) | ≥98% |
| 2-Deoxy-D-ribose | [31170](https://www.sigmaaldrich.com/GB/en/product/sigma/31170) | ≥99.0% |
| 2-Deoxy-L-ribose | [75617](https://www.sigmaaldrich.com/GB/en/product/sigma/75617) | ≥97.0% |
| [D-(+)-Xylose](https://www.sigmaaldrich.com/GB/en/substance/ddxylose1501358866) | X1500 | ≥99% |
| L-(−)-Xylose | [851590](https://www.sigmaaldrich.com/GB/en/product/aldrich/851590) | ≥99% |
| D-(−)-Arabinose | [A3131](https://www.sigmaaldrich.com/GB/en/product/sigma/a3131) | ≥98% |
| [L-(+)-Arabinose](https://www.sigmaaldrich.com/GB/en/substance/ldarabinose150135328370) | [A3256](https://www.sigmaaldrich.com/GB/en/product/sigma/a3256) | ≥99% |
| D-(−)-Fructose | [F0127](https://www.sigmaaldrich.com/GB/en/product/sigma/f0127) | ≥99% |
| L-(+)-Fructose | [31140](https://www.sigmaaldrich.com/GB/en/product/aldrich/31140) | ≥97.0% |
| D-(+)-Glucose | [G8270](https://www.sigmaaldrich.com/GB/en/product/sigma/g8270) | ≥99.5% |
| L-(−)-Glucose | [G5500](https://www.sigmaaldrich.com/GB/en/product/sigma/g5500) | ≥99% |
| D-Alanine | [A7377](https://www.sigmaaldrich.com/GB/en/product/sigma/a7377) | ≥98% |
| L-Alanine | [5129](https://www.sigmaaldrich.com/GB/en/product/sigma/05129) | ≥99.5% |
| L-Valine | [94619](https://www.sigmaaldrich.com/GB/en/product/sigma/94619) | ≥99.5% |
| D-Valine | 855987 | ≥98% |
| D-Leucine | [855448](https://www.sigmaaldrich.com/GB/en/product/aldrich/855448) | 99% |
| L-Leucine | [L8000](https://www.sigmaaldrich.com/GB/en/product/sigma/l8000) | ≥98% |
| D-Isoleucine | [I7634](https://www.sigmaaldrich.com/GB/en/product/sigma/i7634) | ≥98% |
| L-Isoleucine | [I7403](https://www.sigmaaldrich.com/GB/en/product/sigma/i7403) | ≥98.5% |
| [L-Serine](https://www.sigmaaldrich.com/GB/en/substance/lserine1050956451) | [84959](https://www.sigmaaldrich.com/GB/en/product/sigma/84959) | ≥99.5% |
| D-Serine | [S4250](https://www.sigmaaldrich.com/GB/en/product/sigma/s4250) | ≥98% |
| D-Threonine | [T8250](https://www.sigmaaldrich.com/GB/en/product/sigma/t8250) | ≥98% |
| L-Threonine | 89179 | ≥99.5% |
| D-Glutamine | [G9003](https://www.sigmaaldrich.com/GB/en/product/sigma/g9003) | ≥98% |
| L-Glutamine | [G8540](https://www.sigmaaldrich.com/GB/en/product/sigma/g8540) | ≥99% |
| L-Cysteine | [30089](https://www.sigmaaldrich.com/GB/en/product/sigma/30089) | ≥98.5% |
| D-Cysteine | [30095](https://www.sigmaaldrich.com/GB/en/product/aldrich/30095) | ≥99% |
| L-Asparagine | [A0884](https://www.sigmaaldrich.com/GB/en/product/sigma/a0884) | ≥98% |
| D-Asparagine | [A8131](https://www.sigmaaldrich.com/GB/en/product/sigma/a8131) | ≥99% |
| L-Arginine | A8094 | ≥98.5% |
| D-Arginine | [A2646](https://www.sigmaaldrich.com/GB/en/product/sigma/a2646) | ≥98% |
| D-Aspartic acid | [219096](https://www.sigmaaldrich.com/GB/en/product/aldrich/219096) | ≥99% |
| L-Aspartic acid | A9256 | ≥98% |
| D-Glutamic acid | [G1001](https://www.sigmaaldrich.com/GB/en/product/sigma/g1001) | ≥99% |
| L-Glutamic acid | [49449](https://www.sigmaaldrich.com/GB/en/product/sigma/49449) | ≥99.5% |
