# Supplementary material for: Permeability selection of biologically relevant membranes matches the stereochemistry of life on Earth
Source: PLoS Biol. 2025 May 20;23(5):e3003155. doi: 10.1371/journal.pbio.3003155 (PMC12091744; doi:10.1371/journal.pbio.3003155)
Supplement: S2 Table — (DOCX) [file pbio.3003155.s005.docx]

**Table S2. Details of membrane phospholipid compounds used for vesicle synthesis**.

| **Lipid** | **Lipid chemical structure/ IUPAC name** | **Mimic type/Avanti name** | **MW [g/mol]** |
| --- | --- | --- | --- |
| 1. | 2,3-di-O-phytanyl-sn-glycero-1-phosphocholine  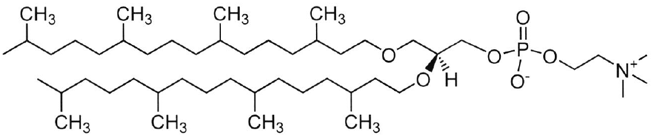 | Archaeal 4ME 16:0 Diether G1PC | 818 |
| 2 | 1,2-dioleoyl-sn-glycero-3-phosphoethanolamine  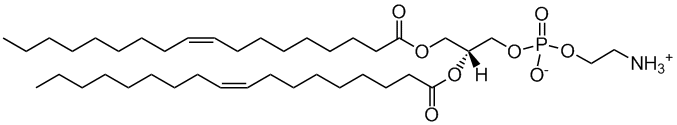 | Bacterial Diester G3PE-PG-CA: 67%: 18:1 (Δ9-Cis) | 744 |
|  | 1,2-dioleoyl-sn-glycero-3-phospho-(1'-rac-glycerol)  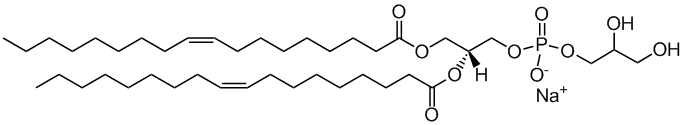 | Diester G3PE23.2%: 18:1 (Δ9-Cis) | 797 |
|  | 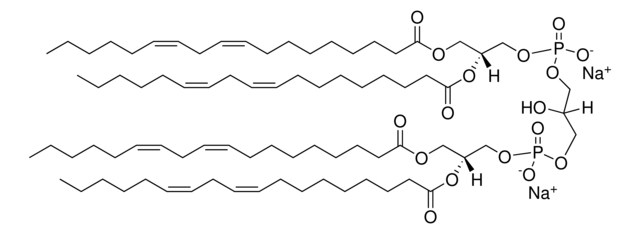1,3-*bis*(*sn*-3'-phosphatidyl)-*sn*-glycerol | Diester G3PG9.8%: Cardiolipin, sodium salt | 1494 |
| 3. | 1,2-di-O-phytanyl-sn-glycero-3-phosphocholine  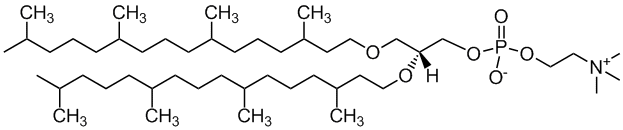 | 4ME 16:0 Diether G3PC | 818 |
