# Supplementary material for: Permeability selection of biologically relevant membranes matches the stereochemistry of life on Earth
Source: PLoS Biol. 2025 May 20;23(5):e3003155. doi: 10.1371/journal.pbio.3003155 (PMC12091744; doi:10.1371/journal.pbio.3003155)
Supplement: S3 Table — (DOCX) [file pbio.3003155.s006.docx]

**Table S3. Parameters used for electroformation of vesicles.**

| **Lipid** | **Frequency [Hz]** | **Amplitude**  **[V]** | **Temp.**  **[°C]** | **Time-rise**  **[min]** | **Time-main**  **[min]** | **Time-fall**  **[min]** |
| --- | --- | --- | --- | --- | --- | --- |
| 1. & 3. | 5 | 3 | 37 | 5 | 120 | 5 |
| 2. | 10 | 1.6 | 37 | 5 | 160 | 5 |
